# Apple endophyte community in relation to location, scion and rootstock genotypes and susceptibility to European canker

**DOI:** 10.1093/femsec/fiab131

**Published:** 2021-10-02

**Authors:** Leone Olivieri, Robert J Saville, Alan C Gange, Xiangming Xu

**Affiliations:** NIAB EMR, New Road, East Malling, Kent ME19 6BJ, UK; Department of Biological Sciences, Royal Holloway, University of London, Egham, Surrey TW20 0EX, UK; NIAB EMR, New Road, East Malling, Kent ME19 6BJ, UK; Agriculture & Horticulture Development Board, Stoneleigh Park, Kenilworth, Warwickshire CV8 2TL, UK; Department of Biological Sciences, Royal Holloway, University of London, Egham, Surrey TW20 0EX, UK; NIAB EMR, New Road, East Malling, Kent ME19 6BJ, UK

**Keywords:** endophytes, apple, meta-barcoding, European apple canker, *Neonectria ditissima*, microbiome

## Abstract

European apple canker, caused by *Neonectria ditissima*, is a severe disease of apple. Achieving effective control is difficult with the currently available pesticides. Specific apple endophytes associated with cultivars may partially contribute to the cultivar response to the pathogen and thus could be used for disease management. We sought to determine whether the overall endophyte community differed among cultivars differing in their susceptibility to *N. ditissima* and to identify specific microbial groups associated with the susceptibility. Using Illumina MiSeq meta-barcoding, we profiled apple tree endophytes in 16 scion–rootstock combinations at two locations and quantified the relative contribution of scion, rootstock and location to the observed variability in the endophyte communities. Endophyte diversity was primarily affected by the orchard location (accounting for 29.4% and 85.9% of the total variation in the PC1 for bacteria and fungi, respectively), followed by the scion genotype (24.3% and 19.5% of PC2), whereas rootstock effects were small (<3% of PC1 and PC2). There were significant differences in the endophyte community between canker-resistant and -susceptible cultivars. Several bacterial and fungal endophyte groups had different relative abundance between susceptible and resistant cultivars. These endophyte groups included putative pathogen antagonists as well as plant pathogens. Their possible ecological roles in the *N. ditissima* pathosystem are discussed.

## Teaser

This study shows that the diversity of fungal and bacterial endophytes in apple trees is influenced by site and tree genotype and associated with susceptibility to *Neonectria ditissima*.

## INTRODUCTION

European apple canker, caused by *Neonectria ditissima* (Tul. & C. Tul.) Samuels & Rossman, is a severe disease of apple in temperate climate regions with frequent rainfall, such as North-Western Europe, the United Kingdom, New Zealand and Chile (Latorre *et al*. 2002; McCracken *et al*. 2003; Weber 2014; Di Iorio *et al*. 2019). Infection takes place at wounds, such as pruning cuts (Xu, Butt and Ridout 1998), leaf scars (Dubin and English 1974), bud scale scars and fruit picking wounds (Amponsah *et al*. 2015). Cankers can girdle shoots and branches, causing loss of fruiting wood, and sometimes kill trees if the cankers are on the main trunk (Swinburne 1975).

Effective canker control is difficult to achieve. Following infection, *N. ditissima* can reside asymptomatically in the plant, and incubation may last from a few months (Amponsah *et al*. 2015; Walter *et al*. 2016) up to 3 years (McCracken *et al*. 2003). For instance, infections may occur in the nursery and remain asymptomatic until after transplanting (McCracken *et al*. 2003; Weber 2014; Saville and Olivieri 2019). Nursery-originated cankers usually affect the main stem and are highly damaging (McCracken *et al*. 2003; Walter *et al*. 2016), especially in modern high-density orchards. Effective canker control in the orchard requires laborious cultural control, e.g. frequent removal of cankers by pruning (Saville and Olivieri 2019) in addition to in-season and postharvest sprays to protect wounds, such as pruning wounds and leaf scars, from infection. A key treatment for canker control, copper oxychloride, has recently been lost through regulation leaving very few effective treatments (Webster *et al*. 2001). Although some apple cultivars show a varying degree of resistance to *N. ditissima* (Garkava-Gustavsson *et al*. 2013; Ghasemkhani *et al*. 2015; Gomez-Cortecero *et al*. 2016), the genetic basis of such resistance is poorly understood, hampering breeding for durable resistance.

Fungal and bacterial endophytes have been studied for their potential application in sustainable crop production (Sturz, Christie and Nowak 2000; Lugtenberg, Caradus and Johnson 2016), because of their ability in improving specific agronomic traits, including disease resistance. Both bacterial and fungal endophytes can act as biocontrol agents against plant pathogens (Ardanov *et al*. 2011; Kurose *et al*. 2012; Busby, Peay and Newcombe 2015; Aziz *et al*. 2016). Endophytic fungi and bacteria might thus provide opportunities for developing novel plant disease management strategies. The community structure of the fungal and bacterial microbiota of apple bark (Arrigoni *et al*. 2018) and xylem (Liu *et al*. 2018) was found to differ among cultivars. It is possible that specific apple endophytes may contribute to the commonly observed cultivar differences in their susceptibility to *N. ditissima*. Several fungal and bacterial apple endophytes were antagonistic to *N. ditissima* in *in vitro* challenge assays (Liu, Ridgway and Jones 2020).

Endophytes in woody hosts are considered predominantly nonsystemic (Saikkonen *et al*. 2004; Moricca and Ragazzi 2008), with distinct microbial communities found in different plant organs (Küngas, Bahram and Põldmaa 2020) and tissues (Bissegger and Sieber 1994) of the same plant. This was shown for apple tree microbiota as well (Liu, Ridgway and Jones 2020). Furthermore, endophyte communities can vary with the development stage of organs (Shade, McManus and Handelsman 2013) and undergo seasonal variation (Ou *et al*. 2019).

In this study, we tested the hypothesis that community structure and relative abundance of bacterial and fungal endophytes of apple significantly differ between canker-resistant and -susceptible cultivars. Samples were collected from five canker-susceptible and three canker-resistant apple cultivars, each grafted on two different rootstocks and planted at two different locations. Total DNA was extracted from leaf scars for amplicon sequencing of bacterial 16S rRNA gene and fungal internal transcribed spacer (ITS) DNA sequences. The relative contribution of design factors (scion, susceptibility level, rootstock and location) to endophyte diversity, as measured by principal components (PCs) and diversity indices, was assessed by analysis of variance (ANOVA). Finally, differential abundance analysis was used to identify endophytes with different relative abundance across canker-resistant and -susceptible cultivars.

## MATERIALS AND METHODS

### Experimental design

Eight apple cultivars (‘Grenadier’, ‘Golden Delicious’, ‘Robusta 5’, ‘Gala’, ‘Braeburn’, ‘Jazz’, ‘Kanzi’, ‘Rubens’) were grafted onto M116 and M9 337 apple rootstocks and planted at two orchards located at two sites in Kent, South-East UK. Therefore, there were 32 treatments: 8 scions × 2 rootstocks × 2 sites (orchards); and four trees per treatment. Within each planting site, there were four blocks; each scion × rootstock treatment was randomly assigned once to each block. Trees in the same block were aligned next to each other in the same row. In addition, scions were grouped in two canker susceptibility levels (susceptible and resistant, respectively) based on the literature. There were five susceptible cultivars, namely ‘Gala’, ‘Braeburn’, ‘Kanzi’, ‘Rubens’ (Weber 2014) and ‘Jazz’ (Berrie 2016); and the remaining three were resistant, namely ‘Grenadier’, ‘Golden Delicious’, ‘Robusta 5’ (Gomez-Cortecero *et al*. 2016). Therefore, the difference among scion genotypes was considered to consist of between the two susceptibility groups and among scions within a susceptibility group. Rootstocks were chosen so that they had different canker susceptibility: M9 337 had higher susceptibility than M116 (Gomez-Cortecero *et al*. 2016).

Trees were grafted at the Frank P Matthews nursery in Worcestershire, UK (Berrington Court, Tenbury Wells) in winter 2016–17 and kept at the nursery until autumn 2018 when they were lifted and placed in a cold store. In early 2018, they were planted at the two orchards: one near Maidstone (Friday St Farm, latitude 51°12′51.6″N, longitude 0°36′41.9″E) and the other near Canterbury (Perry Farm, Wingham; latitude 51°17′01.0″N, longitude 1°14′06.6″E). Orchards were managed according to commercial farm standard practice.

### Sampling

Leaf scar material was sampled in October 2018, at the end of the growing season and before leaf fall had occurred. In this way, we targeted the microorganisms that are most likely to colocalize with *N. ditissima* at this important entry site for the pathogen (Olivieri *et al*. 2021). Temperature and humidity during the week immediately before sample collection were monitored using an EL-USB-2 Data Loggers (Lascar Electronic Ltd, Whiteparish, UK). Four replicates were sampled per each treatment, namely one tree per scion/rootstock combination from each of four blocks, in each orchard.

**Figure 1.**
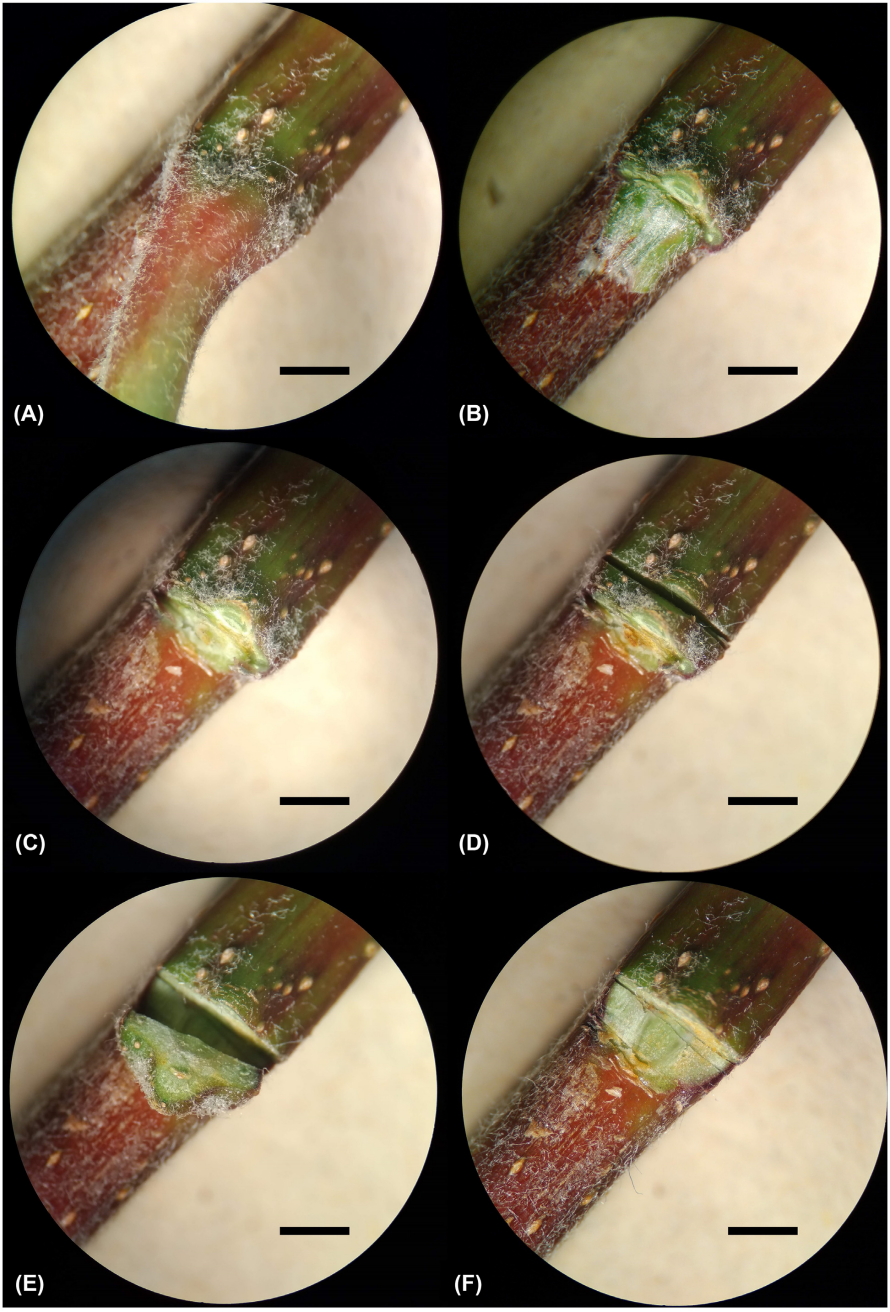
Sampling of a leaf scar from a current season's ‘Gala’ shoot collected in autumn before leaf fall. **(A)** Leaves and petioles were not removed from the shoot until leaf scar dissection was performed. In the lab, **(B)** the petiole was removed with a scalpel blade, **(C)** followed by the axillary bud. Then, **(D)** a transversal cut was performed 1 mm below the leaf scar through the bark layer, **(E)** a tangent cut was made along the stem and a plant tissue sample of ∼4 × 1 mm was excised as a result. The shoot after sample excision is displayed in panel **(F)**. Bar = 2 mm.

On each tree, five 15–20 cm long segments were collected from five shoots (including the main stem) and stored at –20°C until being further processed. On each shoot, three leaf scars were dissected (Fig. 1): (i) the petiole and bud were removed with a scalpel blade, creating a leaf scar; (ii) a transverse cut was performed through the bark layers 1 mm below the leaf scar surface; (iii) a longitudinal cut was then performed between the leaf scar and the stem, from the leaf scar surface towards the base of the shoot. As a result, a 4 × 1 mm fragment of the leaf scar was excised from the shoot. Overall, 15 leaf scars were collected from each tree and pooled. Samples were then stored at –80°C until further preparation for DNA extraction. Samples were freeze-dried over 24 h, flash-frozen in liquid nitrogen and ground into a fine powder in a Geno/Grinder2010 (SPEX SamplePrep, Stanmore, UK) at 1500 rpm for 90 s. Samples were then resuspended in sterile PBS (phosphate buffer saline; Sigma-Aldrich, St Louis, MO) 0.01 M, prepared according to the manufacturer's instructions, in the proportion of 5 ml:1 g dry weight, and shaken in the Geno/Grinder2010 at 1500 rpm for 1 min to ensure thorough homogenization. Samples were stored at –80°C until DNA extraction.

### DNA extraction

DNA was extracted from 100 µl aliquots of resuspended samples using the DNeasy Plant Mini Kit (QIAGEN, Manchester, UK) following the manufacturer's instructions with minor modifications, notably: at step 2, the sample was incubated for 15 min in 65°C; at step 11 the DNA was eluted in 100 μl buffer AE. DNA quality and quantity were checked spectrophotometrically (NanoDrop 1000, Thermo Fisher Scientific, Waltham, MA).

### Library preparation

The amplicon library was prepared as described in Tilston *et al*. (2018). The primers EkITS1F (5′-CTT GGT CAT TTA GAG GAA GTA A-3′) (Gardes and Bruns 1993) and Ek28R (5′-AT ATG CTT AAG TTC AGC GGG-3′) (corresponding to 3126T in Sequerra *et al*. 1997 and HC2 in Navajas *et al*. 1994) were used to amplify the nonoverlapping variable ITS1 and ITS2 regions in the fungal genome. These primers were chosen because they had been shown to give better amplification when combined with the Illumina adapter (Xu *et al*.^2015^). The primers Bakt_341F (5′-CCT ACG GGN GGC WGC AG-3′) and Bakt_805R (5′-GGA CTA CHV GGG TAT CTA ATC C-3′) were used to amplify the V3–V4 variable region of the 16S rRNA gene. These primers were chosen because they were shown by Herlemann *et al*. (2011) to match perfectly over 90% of the respective bacterial 16S rRNA gene sequences in the Ribosomal Database Project (RDP) release 10.25 (Cole *et al*. 2009); and have been successfully used to characterize bacterial communities in rhizosphere soil samples (Tilston *et al*. 2018). Both primer sets were modified at the 5′ end with adaptors: TCG TCG GCA GCG TCA GAT GTG TAT AAG AGA CAG (forward primer adaptor) and GTC TCG TGG GCT CGG AGA TGT GTA TAA GAG ACA (reverse primer adaptor). All ITS and 16S rRNA gene polymerase chain reactions (PCRs) were run in triplicate per each sample and technical replicates were then pooled. PCRs were carried out in a T100 Thermal Cycler (Bio-Rad Laboratories, Hercules, CA) with 4 ng of template DNA in a total volume of 13 µl. All reactions contained 0.05 µl MolTaq (Molzym, Bremen, Germany), 1.25 µl MolTaq buffer 10×, 1.0 µl dNTPs 2.5 mM, 0.25 µl MgCl_2_ 100 mM and 1.25 µl of each primer 2 µM. ITS PCR conditions were as follows: 95°C for 5 min, followed by 30 cycles at 94°C for 30 s, 52°C for 45 s and 72°C for 60 s, with a final extension at 72°C for 7 min. 16S rRNA gene PCR conditions were as above, but the annealing temperature was 55°C and the number of cycles was 25. PCR products were viewed on 1.5% agarose before proceeding to clean-up and indexing.

### PCR clean-up, sample indexing and sequencing

Equal volumes of fungal and bacterial amplicons were pooled according to individual plant samples. Then PCR clean-up was performed with the Agencourt AMPure XP kit (Beckman Coulter, Brea, CA) following the manufacturer's instructions. Sample indexing was carried out by ligating Illumina compatible adapters using the Nextera^®^ XT Index Kit v2 (Illumina, San Diego, CA) and the KAPA HiFi HotStart ReadyMix kit (Roche, Basel, Switzerland). All reactions were carried out in a 50 µl volume with 25 µl KAPA reaction mix 2×, 5 µl of both forward and reverse index primers and 5 µl of cleaned-up DNA sample. Index PCR clean-up was performed with the Agencourt AMPure XP kit, then quantity and quality of the cleaned-up DNA were checked using a NanoDrop 1000 spectrophotometer (Thermo Fisher Scientific) and DNA was diluted to 10 ng/µl. Subsequently, DNA concentration was checked with a Qubit2.0 fluorometer (Life Technologies, Carlsbad, CA) and samples were pooled into a 4 nM library. The library was denatured with 0.1 M NaOH and diluted to 12 pM according to the manufacturer's protocol (16S Metagenomic Sequencing Library Preparation protocol, Illumina [2019]), then it was combined with a denatured PhiX library (PhiX Control v3, Illumina) at an equimolar concentration at a rate of 20% v/v to increase heterogeneity of the samples. Sequencing was carried out on an Illumina MiSeq instrument using the 300 bp paired-end protocol (16 indexing cycles and 301 sequencing cycles, total 618 cycle run) and the MiSeq v3 reagent cartridge and kit (Illumina).

### Bioinformatic analysis of sequence reads

#### Sequence processing

Sequence processing was performed with the methods/pipelines described by Deakin *et al*. (2018). FASTQ reads were first demultiplexed into bacterial (16S rRNA gene) and fungal (ITS) datasets based on their primer sequences. Reads with any ambiguous positions in the primer region or nonmatching forward and reverse primers were discarded. Demultiplexed sequences were then filtered according to length and quality criteria to generate the operational taxonomic units (OTUs).

#### Generation of OTUs and OTU tables

Paired-end 16S rRNA gene reads had an overlap of ∼160 nucleotides (NT) and were merged with a minimum merged sequence length of 300 NT and 5% maximum difference in overlap. Merged reads shorter that 300 NT or containing adapters contamination were excluded from further analysis, and the remaining merged reads were trimmed by 17 bp at the left end and 21 bp at the right end, to remove forward and reverse primers respectively. Merged sequences were then filtered for quality with maximum expected error threshold of 0.5 per sequence (Edgar and Flyvbjerg 2015) before OTU clustering. The expected distance between forward and reverse fungal ITS primer was greater than the maximum MiSeq merged read length (600 bp), hence reads could not be merged. Only forward reads (ITS1) were used in the analysis. Sequences were truncated at 250 NT, all reads shorter than 250 NT or containing adapters contaminations were discarded, then the remaining reads were truncated by 22 bp at the left end to remove the forward primer sequence. Reads were quality filtered with maximum expected error threshold of 1 per sequence before OTU clustering. Filtered 16S rRNA gene and ITS reads were dereplicated to obtain unique sequences, then unique sequences with fewer than 2 occurrences overall (i.e. singletons) were discarded for the purpose of generating OTUs. The remaining unique sequences were clustered into OTUs at the level of 97% similarity and a representative sequence was generated for each OTU. Chimeras were identified and removed by the clustering algorithm. Finally, all unfiltered sequences were aligned with the OTU representative sequences at the level of 97% similarity to generate ITS and 16S rRNA gene OTU frequency tables (i.e. OTU table).

#### Assignment of taxonomic rank

Taxonomic predictions were made with the SINTAX algorithm (Edgar 2016) by aligning the OTU representative sequences with the reference databases and ‘RDP training set v16’ for the 16S rRNA gene (Cole *et al*. 2014) and ‘UNITE v8.0’ for the ITS (Nilsson *et al*. 2019; UNITE Community 2019).

### Statistical analyses

#### Library normalization

All statistical analyses were carried out with R 3.6.1 (R Core Team 2019). To minimize the effects of sampling, including sequencing depth and OTU composition, OTU count data were normalized before statistical analysis for library size using the median-of-ratio method implemented in DESeq2 (Love, Huber and Anders 2014). Before principal component analysis (PCA), normalized counts were further transformed with the DESeq2 variance stabilizing transformation (VST) method to make data approximately homoscedastic (Anders and Huber 2010). After normalization and transformation, chloroplast and mitochondria V4 rDNA (identified by BLASTn; Zhang *et al*. 2000) were excluded from all downstream analyses.

#### ANOVA of PCA scores

PCA was carried out on the VST transformed data. ANOVA was then performed on the first four PCs to assess the contribution of experimental factors to the proportion of variance in the PC scores. Experimental factors included site, blocks within each site, canker susceptibility, between scion genotypes within each susceptibility level, rootstock genotype, and the scion and rootstock interaction.

#### Alpha and beta diversity

Normalized OTU counts were rounded prior to calculation of alpha (α) diversity indices to allow computation of indices based on species singletons and doubletons. Species richness indices (observed species and Chao1) and diversity indices (Shannon and Simpson) were calculated with the R package phyloseq v1.30.0 (McMurdie and Holmes 2013). The effects of experimental factors on these alpha diversity indices were analysed by permutational ANOVA, as implemented in the R package lmPerm v2.1.0 (Wheeler and Torchiano 2016). Statistical significance was estimated by 5000 permutations based on sequential sum of squares. For beta (β) diversity, Bray–Curtis indices were calculated from normalized OTU counts. Nonmetric multidimensional scaling (NMDS) of the Bray–Curtis indices was carried out with the R package Vegan 2.5-6 (Oksanen *et al*. 2019). Permutational multivariate analysis of variance (PERMANOVA), as implemented in Vegan 2.5-6 in the function ‘adonis’, was carried out to assess the effects of experimental factors on the Bray–Curtis indices. Statistical significance was estimated by 9999 permutations based on sequential sum of squares.

#### Differential OTU abundance

Comparison of relative abundance of individual OTUs between the resistant and susceptible cultivars was performed in R with DESeq2. DESeq2 analyses raw OTU counts and compares species relative abundances between groups using generalized linear modelling, assuming a negative binomial distribution for residuals (Anders and Huber 2010; Love, Huber and Anders 2014). To account for library size, count data were normalized with the median-of-ratios method. Prior to differential abundance analysis, DESEq2 performs independent filtering of OTUs according to several criteria, including the overall abundance level and the variance in abundance across samples. DESeq2 uses a Wald test to test for significance of differentially abundant OTUs with the Benjamini–Hochberg (BH) method (Benjamini and Hochberg 1995) used to adjust the p-value.

Before running DESeq2, the block factor was recoded to include the site factor, resulting in a block factor with eight levels that accounted for all differences between and within sites. The models fitted in DESeq2 were chosen based on the results of overall community structure analysis (ANOVA on PCs and PERMANOVA on Bray–Curtis index), and were: (i) scion, recoded block and rootstock for bacteria and (ii) scion, recoded block, rootstock and scion and rootstock interaction for fungi. For fungi, the maximum number of iterations of the Wald test for GLM coefficients was increased to 5000 to allow for convergence of the coefficient vector. Difference between resistant and susceptible genotypes was statistically tested in DESeq2 by a single contrast: (‘Robusta 5’, ‘Golden Delicious’ and ‘Grenadier’) against (‘Rubens’, ‘Gala’, ‘Kanzi’, ‘Jazz’ and ‘Braeburn’). Furthermore, the difference between ‘Robusta 5’ and all other genotypes (‘Golden Delicious’, ‘Grenadier’, ‘Rubens’, ‘Gala’, ‘Kanzi’, ‘Jazz’, ‘Braeburn’) was tested by a single contrast, since the preliminary analysis suggested distinct differences of ‘Robusta 5’ from the other cultivars. Statistical significance was determined at the 5% level (BH adjusted). SINTAX taxonomic annotations for differentially abundant OTUs were made at the lowest possible rank when (i) the SINTAX confidence value was at least 0.7, or (ii) BLASTn 2.10.1+ (Zhang *et al*. 2000) confirmed SINTAX prediction against NCBI's nonredundant nucleotide collection (nt). BLASTn hits were accepted when they proved unambiguous based on the query coverage (min 100%), % identity and *e*-value. Taxonomic predictions were accepted up to the taxon level on which there was agreement between BLASTn and SINTAX. Putative lifestyle and ecological functions were obtained from available peer-reviewed literature.

## RESULTS

### Sequence quality and generation of OTUs

After quality and length filtering, 6 332 704 bacterial 16S rRNA gene reads were obtained from 128 samples. Of these, 84 227 were identified as unique sequences and clustered into 115 OTUs, including chloroplast and mitochondrial DNA sequences. Unfiltered 16S rRNA gene reads were aligned to the OTUs, ranging from 26 901 to 134 339 per sample. After removal of chloroplast and mitochondrial DNA sequences, a total of 32 719 aligned reads were left (∼0.4% of total unfiltered reads), ranging from 13 to 2701 per sample. Sequence depth was checked after removal of chloroplast and mitochondrial OTUs, and 23 samples were excluded from downstream analysis because of inadequate sequence depth. Accumulation curves for 16S rRNA gene reads are available in Fig. S1 (Supporting Information). Of these 23 samples, 15 were from resistant genotypes (8 ‘Robusta 5’, 4 ‘Golden Delicious’ and 1 ‘Grenadier’) and 8 were from susceptible genotypes (4 ‘Gala’, 3 ‘Rubens’ and 1 ‘Braeburn’); 13 and 10 were from trees grafted to M116 and M9 337 rootstocks, respectively; and 18 and 5 from Canterbury and Maidstone orchards, respectively. Overall, post removal of chloroplast and mitochondrial sequences and of samples with insufficient sequence depth, 79.6% of bacterial reads were captured by the 10% most abundant 16S rRNA gene OTUs (Fig. S2, Supporting Information).

After quality and length filtering, 2 545 933 fungal ITS reads were obtained from the samples. Of these, 50 470 were identified as unique and clustered into 706 OTUs. The number of unfiltered reads that were aligned to these OTUs ranged from 2510 to over 50 000 per sample. Sequencing depth was sufficient for all samples. Accumulation curves for ITS reads are available in Fig. S3 (Supporting Information). In all samples collected, only a few OTUs had high read counts. Overall, 99.0% and 86.4% of fungal reads were captured by the 10% and 2% most abundant ITS OTUs, respectively (Fig. S4, Supporting Information).

### ANOVA of PCA scores

**Figure 2.**
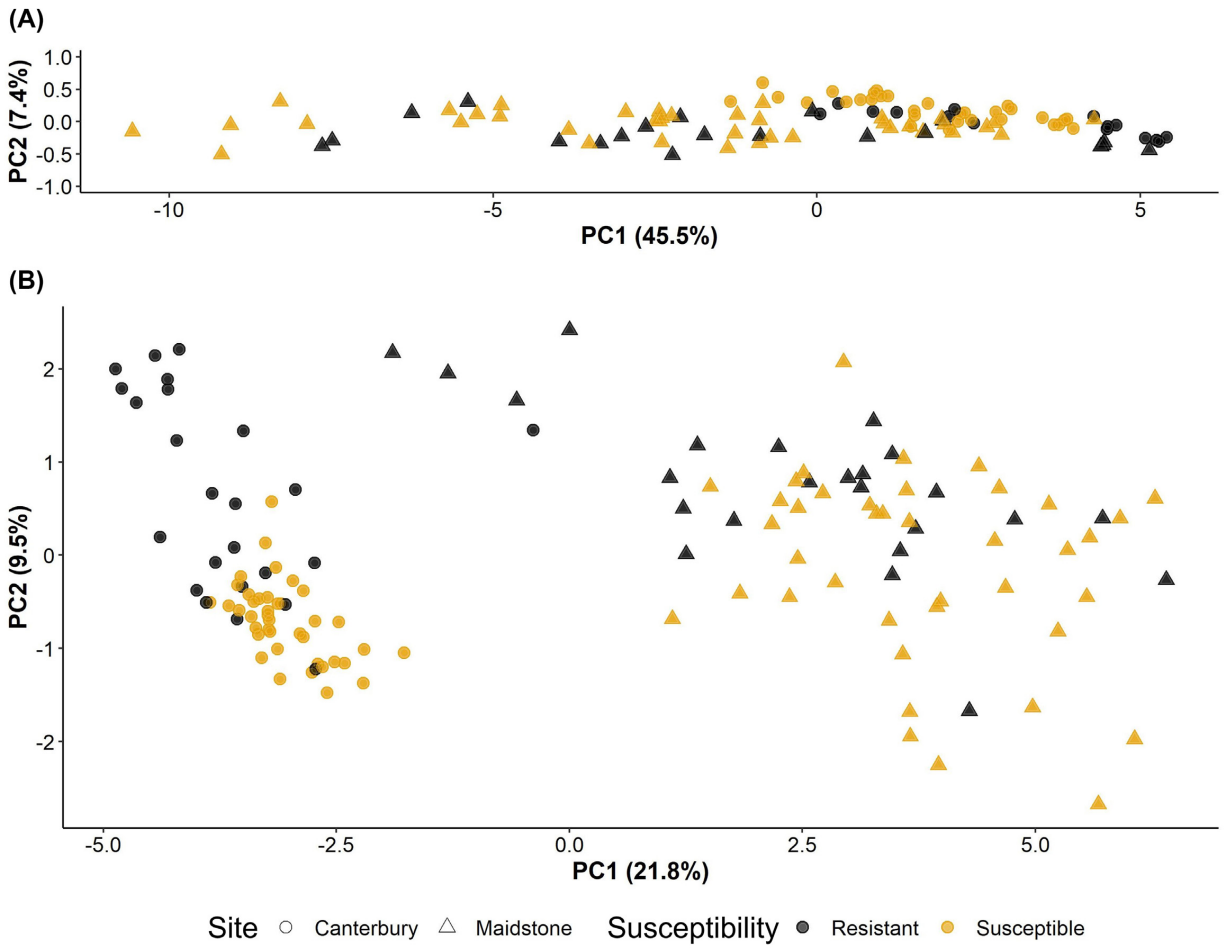
PCA of **(A)** bacterial and **(B)** fungal OTUs; in both plots, PC1 vs PC2 are shown and the percentage of variance explained by each PC is reported in brackets. PC axes were rescaled by multiplying PC scores by the percentage of variance explained by the respective PC to reflect equal distances between the two directions for ease of interpretation. Points are biological replicates, point shape represents the site (Maidstone or Canterbury) and point colour represents the susceptibility level of the cultivar (resistant or susceptible).

Figure 2 shows the first two PC scores of VST-transformed bacterial and fungal OTU counts and Table 1 gives the ANOVA results of PC1–PC4. For bacteria, the first four PCs accounted for 62.0% of the total variance (45.5%, 7.4%, 5.1% and 4.0% for PC1–PC4, respectively). The location accounted for 29.4% and 20.5% (*P* < 0.001) of the total variability in PC1 and PC2, respectively, whereas the scion genotype within the susceptibility group accounted for ∼24.0% of the total variability in both PC1 and PC2. The susceptibility level had significant effects on both PC1 (11.7%, *P* < 0.001) and PC2 (2.0%, *P* < 0.05). PC1 was also affected by blocks within a site (7.8%, *P* < 0.01) and rootstock genotype (2.8%, *P* < 0.05). PC3 was affected by location, blocks within site and scion genotypes within the susceptibility group whereas PC4 was affected by the susceptibility level and the scion genotype within each susceptibility group. The interaction between scion and rootstock genotypes was not significant on all four PCs.

**Table 1.** Results of ANOVA on bacterial and fungal PCs 1 through 4, showing the percentage of variability (%var) in PC scores accounted for by design factors and the respective significance level (*p*); the effect of the scion × rootstock interaction was never significant and therefore is not reported.

		Site		Block within site		Susceptibility		Scion within susceptibility		Rootstock		Residuals
PC	% total variance	% var	*p*	% var	*p*	% var	*p*	% var	*p*	% var	*p*	% var
*Bacteria*												
PC1	45.5	29.4	<0.001	7.8	0.007	2.0	0.029	23.4	<0.001	2.8	0.011	33.2
PC2	7.4	20.5	<0.001	2.7	0.438	11.7	<0.001	24.3	<0.001	0.2	0.523	37.2
PC3	5.1	6.6	0.004	16.5	0.002	0.7	0.346	13.4	0.01	0.2	0.624	60.4
PC4	4.0	1.3	0.234	2.6	0.801	4.7	0.023	15.2	0.013	0.2	0.668	71.3
*Fungi*												
PC1	21.8	85.9	<0.001	1.3	0.004	1.7	<0.001	4	<0.001	<0.1	0.468	6.6
PC2	9.5	3.9	0.002	6.5	0.011	27.3	<0.001	19.5	<0.001	1.0	0.096	38.8
PC3	3.4	0.9	0.163	15.9	<0.001	5.0	0.002	25	<0.001	0.4	0.362	50.3
PC4	2.5	0.8	0.232	12.2	0.002	<0.1	0.696	26.4	<0.001	7.0	0.258	57.2

For fungi, the first PCs accounted for 37.2% of the total variance (21.8%, 9.5%, 3.4% and 2.5% for PC1–PC4, respectively) (Table 1). PC1 was mainly influenced by the location (85.9%, *P* < 0.001), whereas PC2 was mainly influenced by the susceptibility level (27.3%, *P* < 0.001) and scion genotypes within a susceptibility group (19.5%, *P* < 0.001). Blocks within a site had significant effects on PC1 (1.3%, *P* > 0.01) and PC2 (6.5%, *P* > 0.05). PC3 was affected by blocks within a site, the susceptibility level and scion genotypes within a susceptibility group; whilst PC4 was affected by blocks within site and scion genotypes within a susceptibility group. PC1–PC4 were not significantly affected by the rootstock and its interaction with scion genotypes.

### Diversity indices

A summary of α diversity indices for bacteria and fungi data is given in Table S1 and Fig. S5 (Supporting Information). Among the indices, the observed OTU number was most affected by experimental factors (Table 2). For bacteria, the number of OTUs was primarily influenced by location, followed by scion genotypes within a susceptibility level, blocks within a site, rootstock and canker susceptibility (resistant = 21.6, susceptible = 24.8), whereas the scion and rootstock interaction was not significant. For fungi, the number of OTUs was primarily influenced by location, followed by blocks within a site, scion within a susceptibility level, canker susceptibility (resistant = 97.6, susceptible = 128.7) and finally the scion and rootstock interaction. The effect of canker susceptibility was greater for fungi (14.4%, *P* < 0.001) than for bacteria (1.5%, *P* < 0.05). The bacterial Shannon indices were primarily affected by scion genotypes within a susceptibility level, followed by site, canker susceptibility (resistant = 1.97 and susceptible = 2.29) and blocks within a site. The bacterial Simpson index was primarily affected by scions within a susceptibility level, followed by canker susceptibility (resistant = 0.74 and susceptible = 0.83), blocks within a site and site. Shannon and Simpson indices for fungi were primarily affected by scions within a susceptibility level, followed by blocks within a site, whereas effects of other factors on the two indices were statistically not significant.

**Table 2.** Percentage of variability (%var) in alpha (Shannon, Simpson, observed species and Chao1) and beta (Bray–Curtis) diversity indices accounted for by design factors and interactions and the respective significance level (*p*).

	Site		Block within site		Susceptibility		Scion within susceptibility		Rootstock		Scion:rootstock		Residuals
Measure^a^	%var	*p*	%var	*p*	%var	*p*	%var	*p*	%var	*p*	%var	*p*	%var
*Bacteria*													
Observed	38.0	<0.001	7.6	0.004	1.5	0.046	18.2	<0.001	1.9	0.033	1.9	0.584	30.8
Chao1	35.5	<0.001	6.4	0.067	0.4	0.228	16.0	0.001	0.0	0.619	3.3	0.484	38.5
Shannon	20.3	<0.001	6.6	0.013	9.1	<0.001	23.2	<0.001	0.1	0.745	3.3	0.490	37.4
Simpson	6.5	<0.001	7.6	0.038	10.8	<0.001	31.3	<0.001	0.0	0.726	4.0	0.330	39.9
Bray–Curtis	12.3	<0.001	7.5	<0.001	3.4	<0.001	20.6	<0.001	1.3	0.045	4.2	0.510	50.7
*Fungi*													
Observed	29.7	<0.001	17.8	<0.001	14.4	<0.001	16.0	<0.001	0.4	0.092	2.6	0.045	19.1
Chao1	2.8	0.031	3.8	0.494	7.9	0.000	9.3	0.024	0.0	1.000	7.4	0.115	68.8
Shannon	0.3	0.474	10.4	0.012	3.0	0.062	17.8	<0.001	0.4	1.000	2.3	0.775	65.8
Simpson	0.0	0.843	15.3	<0.001	0.0	0.922	19.5	<0.001	0.3	0.396	2.3	0.878	62.6
Bray–Curtis	36.6	<0.001	5.2	<0.001	6.2	<0.001	14.1	<0.001	0.4	0.278	3.5	0.039	34.0

a Results from permutational ANOVA of alpha diversity indices or PERMANOVA of Bray–Curtis dissimilarity matrix.

**Figure 3.**
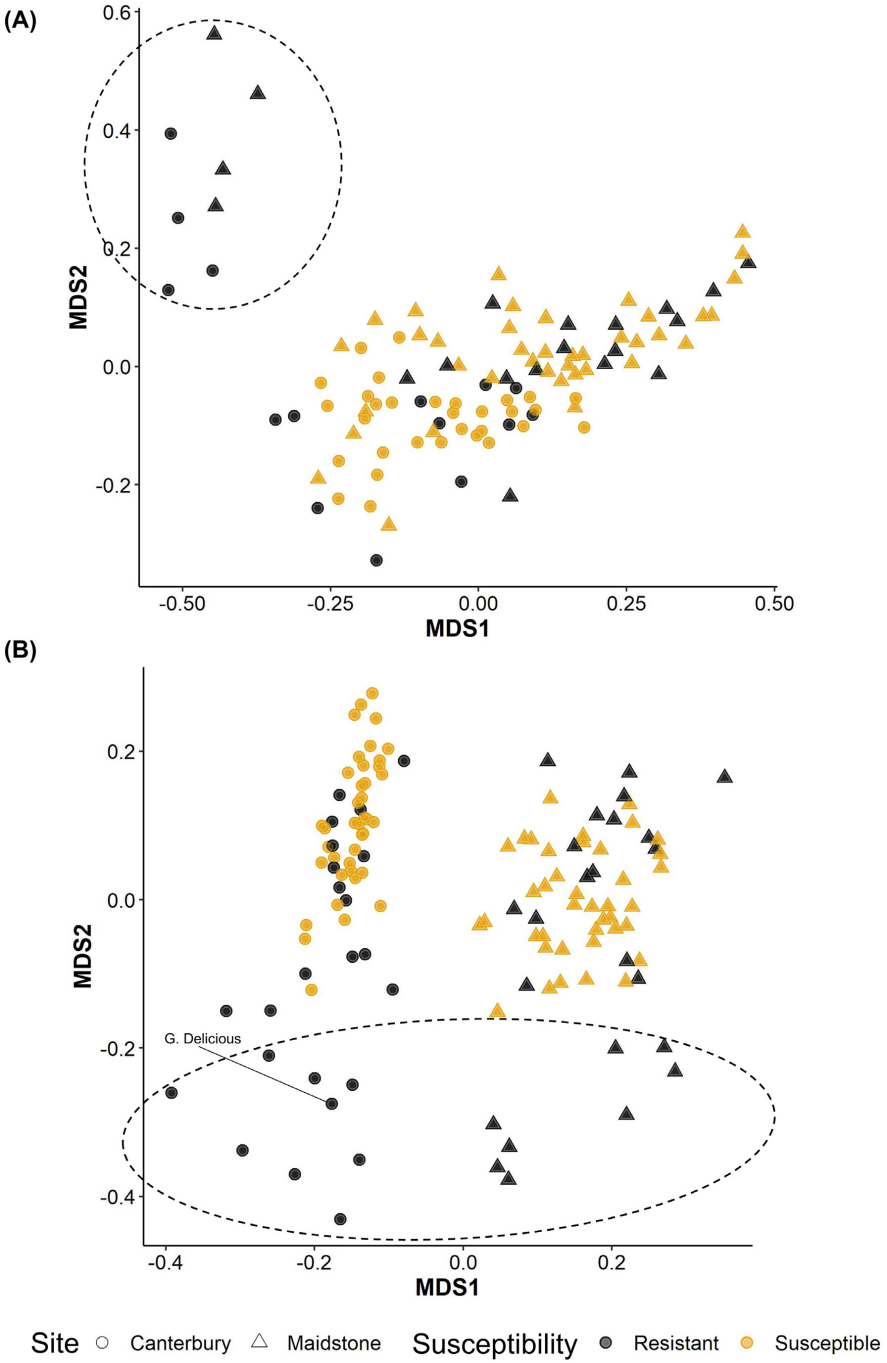
Structure of microbial communities: NMDS ordination of **(A)** bacterial and **(B)** fungal Bray–Curtis dissimilarity matrices. Stress: (A) 0.123, (B) 0.134. Points are biological replicates, point shape represents site (Maidstone or Canterbury) and point colour represents canker susceptibility level of the cultivar (resistant or susceptible). Points enclosed in dashed line are ‘Robusta 5’ replicates, except for the point labelled as ‘Golden Delicious’ in panel (B).

NMDS ordination was used to visualize bacterial and fungal β diversities (Fig. 3). For bacteria, no obvious clustering was observed in relation to location and the susceptibility level (Fig. 3A). However, the scion genotype ‘Robusta 5’ clustered away from all the other genotypes. For fungi, samples were clustered primarily based on the location (Fig. 3B). As for bacteria, the scion ‘Robusta 5’ clustered away from all the other genotypes. Adonis analysis (Table 2) showed that bacterial community structure, as measured by Bray–Curtis indices, was mainly affected by scions within the susceptibility level, followed by site, blocks within a site and canker susceptibility (*P* < 0.001). For fungi, it was primarily affected by site, followed by scions within a susceptibility level, canker susceptibility and blocks within a site (*P* < 0.001). Rootstocks had significant albeit small effects on bacterial β diversity (1.3% explained variability, *P* < 0.05), whereas the scion and rootstock interaction was not significant. For fungi, rootstock effect was not significant (*P* = 0.278) but the scion and rootstock interaction had a significant effect (3.5% explained variability, *P* < 0.05). A greater proportion of variability in the β diversity indices of bacterial community was unaccounted for (50.7%) compared with the fungal community (34.0%).

### Differential analysis of OTU relative abundance

The number of bacterial and fungal OTUs before and after DESeq2 independent filtering, as well as the number of OTUs with significantly differential relative abundance between resistant and susceptible scion genotypes, are given in Table 3. Canker-resistant and -susceptible cultivars differed significantly in the relative abundance of 19 bacterial (Table 4) and 31 fungal OTUs (Table 5). The best prediction of taxonomy for differentially abundant OTUs is available in Table S2 (Supporting Information). A plot of the log_2_ of the mean normalized OTU counts over the log_2_ of the fold change (log_2_FC) estimate is given for these OTUs (Fig. S6, Supporting Information).

**Table 3.** Summary of bacterial and fungal OTUs before filtering, after DESeq2 independent filtering and with significant differential relative abundance in resistant apple genotypes compared with susceptible genotypes via DESeq2 analysis.

	Number of OTUs	
	Bacteria^a^	Fungi
Before DESeq2 filtering	113	706
After DESeq2 independent filtering	30 (26.5%)	213 (30.2%)
With Benjamini–Hochberg adjusted *P*-value < 0.05	19 (16.8%)	31 (4.4%)

a Bacterial OTUs after removal of chloroplast and mitochondrial OTUs.

**Table 4.** Bacterial OTUs with differential relative abundance in resistant apple genotypes compared with susceptible genotypes, and putative lifestyle and ecology. No OTUs were found with higher relative abundance in resistant genotypes. Negative log_2_FC corresponds to higher relative abundance in susceptible genotypes. Taxonomic annotations were done with UPARSE using the UNITE database and checked with BLASTn on NCBI's nonredundant nucleotide collection (nr/nt). *P*-value was corrected for the false discovery rate with the Benjamini–Hochberg (BH) method and statistical significance was determined at the 5% level. FC = fold change; SE = standard error; *P*-value (BH) = Benjamini–Hochberg corrected *P*-value.

UNITE taxon	Mean normalized count^a^	Log_2_FC ± SE	*P*-value (BH)	Putative lifestyle and ecology
*Sphingomonas* sp. (Sphingomonadaceae)	80.41	−5.17 ± 0.74	<0.001	Widely distributed; biological control activity against fungal pathogens of wheat (Wachowska *et al*. 2013); promoter of plant growth (Asaf *et al*. 2020)
*Sphingomonas* sp.	56.43	−4.01 ± 0.65	<0.001	
*Curtobacterium* sp. (Microbacteriaceae)	26.22	−3.83 ± 0.72	<0.001	Cosmopolitan genus with a variety of ecological roles (Chase *et al*. 2016 ); includes plant pathogenic species (Osdaghi *et al*. 2015), as well as species with beneficial effects such as plant growth promotion (Sturz *et al*. 1997) or disease reduction (Lacava *et al*. 2007)
*Massilia* sp. (Oxalobacteriaceae)	16.64	−3.89 ± 0.67	<0.001	
*Pseudomonas* sp. (Pseudomonadaceae)	16.08	−1.45 ± 0.52	0.012	Widely distributed; includes species pathogenic on apple (Kennelly *et al*. 2007), as well as species producing antifungal compounds (Ligon *et al*. 2000)
*Pseudomonas* sp.	13.63	−3.41 ± 0.59	<0.001	
*Hymenobacter* sp. (Hymenobacteraceae)	13.49	−14.12 ± 1.06	<0.001	
*Hymenobacter* sp.	8.48	−3.34 ± 1.10	0.007	
*Hymenobacter* sp.	7.94	−4.33 ± 1.09	<0.001	
*Frondihabitans* sp. (Microbacteriaceae)	7.13	−2.33 ± 0.81	0.010	
*Methylobacterium* sp. (Methylobacteriaceae)	6.98	−1.84 ± 0.84	0.049	Includes species with beneficial effects on plants, including promotion of plant growth (Dourado *et al*. 2015) and antagonism of plant pathogenic fungi *in vitro* and *in vivo* (Grossi *et al*. 2020)
*Hymenobacter* sp.	6.41	−2.32 ± 1.07	0.049	
Rhizobiales	5.82	−2.73 ± 0.92	0.008	
*Massilia* sp.	5.01	−3.78 ± 1.23	0.007	
*Massilia* sp.	3.93	−2.09 ± 0.90	0.038	
*Methylobacterium* sp.	3.88	−11.37 ± 1.19	<0.001	
*Hymenobacter* sp.	3.22	−2.66 ± 1.23	0.049	
*Rathayibacter* sp. (Microbacteriaceae)	1.36	−2.51 ± 1.08	0.038	
*Hymenobacter* sp.	1.08	−13.83 ± 2.04	<0.001	

a Mean normalized count across resistant and susceptible cultivars.

**Table 5.** Fungal OTUs with differential relative abundance in resistant apple genotypes compared with susceptible genotypes, and putative lifestyle and ecology. Positive log_2_FC corresponds to higher relative abundance in resistant genotypes; negative values correspond to higher relative abundance in susceptible genotypes. Taxonomic annotations were done with UPARSE using the UNITE database and BLASTn on NCBI's nonredundant nucleotide collection (nr/nt). *P*-value was corrected for the false discovery rate with the Benjamini–Hochberg (BH) method and statistical significance was determined at the 5% level. FC = fold change; SE = standard error; *P*-value (BH) = Benjamini–Hochberg corrected *P*-value.

Taxon^a^	Mean normalized count^b^	Log_2_FC ± SE	*P*-value (BH)	Putative lifestyle and ecology
***Higher relative abundance***				
*Aureobasidium* sp. (Aureobasidiaceae)	1785.02	1.51 ± 0.38	0.001	Yeast-like fungus with antagonistic activity against plant-pathogenic bacteria and fungi in the field and postharvest (Mari *et al*. 2012; Freimoser *et al*. 2019), and plant growth-promoting activity (Di Francesco *et al*. 2021)
*Rhodotorula* sp. (Sporidiobolaceae)	850.58	3.19 ± 0.45	<0.001	Pigmented yeast genus, including plant growth promoting species (Firrincieli *et al*. 2015) and biocontrol agents (Li *et al*. 2011)
Fungi	726.54	1.18 ± 0.26	<0.001	
*Stemphylium* sp. (Pleosporaceae)	214.19	1.63 ± 0.30	<0.001	*S. vesicarium* causes blossom-end rot of apple (Weber and Dralle 2013); *S. botryosum* and *S. herbarum* cause postharvest rot of apple (Behr 1960; Jijakli and Lepoivre 2004)
*Kalmanozyma* sp. (Ustilaginaceae, possibly *K. fusiformata*)	57.42	3.18 ± 1.15	0.040	*K. fusiformata* is a ‘killer yeast’ (Klassen *et al*. 2017), producing an antifungal glycolipid (Golubev, Kulakovskaya and Golubeva 2001; Kulakovskaya *et al*. 2007)
Dothideomycetes	14.27	1.60 ± 0.51	0.015	
Entylomatales	8.03	3.49 ± 1.11	0.015	
*Dissoconium* sp. (Dissoconiaceae, possibly *D. eucalypti*)	3.27	3.52 ± 1.07	0.001	Species in this genus were co-isolated with other fungal species from leaf spots, sooty blotch and flyspeck of fruits (Li *et al*. 2012); also found on a wide range of host plants with different ecological roles, from plant pathogens to mycoparasites (Crous *et al*. 1999; Kiss 2003)
***Lower relative abundance***				
*Vishniacozyma* sp. (Bulleribasidiaceae)	1185.27	−3.20 ± 0.66	<0.001	Basidiomycetous yeast, found in soil (Mašínová *et al*. 2017) as well as different plant species (Ricks and Koide 2019), including apple (Bösch *et al*. 2021)
*Filobasidium* sp. (Filobasidiaceae, possibly *F. floriforme*)	506.17	−1.67 ± 0.45	0.003	Basidiomycetous yeast, found in different plant species (Ricks and Koide 2019), as well as in apple leaves (Glushakova and Kachalkin 2017a) and bark (Arrigoni *et al*. 2020)
*Filobasidium* sp. (possibly *F. wieringae*)	460.93	−2.45 ± 0.50	<0.001	
*Dioszegia* sp. (Bulleribasidiaceae, possibly *D. hungarica*)	416.16	−1.59 ± 0.56	0.031	Basidiomycetous yeast with hypothesized mycoparasitic lifestyle (Begerow *et al*. 2017); found in different plant species (Ricks and Koide 2019), including grape (Nemcová *et al*. 2015) and apple (Abdelfattah *et al*. 2016)
*Filobasidium* sp. (possibly *F. floriforme*)	394.16	−1.29 ± 0.42	0.017	
*Vishniacozyma* sp. (possibly *V. carnescens*)	221.74	−2.68 ± 0.66	0.001	
*Gelidatrema* sp. (Phaeotremellaceae, possibly *G. spencermartinsiae*)	155.09	−3.66 ± 0.68	<0.001	Basidiomycetous yeast, reported in apple fruits (De García *et al*. 2010; Bösch *et al*. 2021)
Basidiomycota	149.67	−1.69 ± 0.57	0.023	
*Filobasidium* sp. (possibly *F. chernovii)*	137.28	−2.47 ± 0.48	<0.001	
*Genolevuria* sp. (Bulleraceae, possibly *G. amylolytica*)	79.86	−3.58 ± 0.71	<0.001	Basidiomycetous yeast, found in decaying wood (Behnke-Borowczyk *et al*. 2018); also found in significant association with *Hymenoscyphus fraxineus* (ash dieback) in infected plant material (Griffiths *et al*. 2019)
Fungi	45.96	−2.52 ± 0.82	0.017	
Tremellomycetes	41.46	−4.60 ± 0.79	<0.001	
*Bulleromyces* sp. (Tremellaceae, possibly *B. albus*)	34.17	−2.08 ± 0.60	0.005	Basidiomycetous yeast with antifungal activity (Golubev *et al*. 1997); reported in apple bark (Arrigoni *et al*. 2020)
*Dioszegia* sp.	22.62	−3.30 ± 0.91	0.003	
Fungi	17.54	−2.80 ± 0.79	0.004	
*Filobasidium* sp. (possibly *F. oeirense*)	14.68	−3.36 ± 0.82	0.001	
*Papiliotrema* sp. (Rhynchogastremataceae, possibly *P. frias*)	11.07	−3.59 ± 0.84	<0.001	Basidiomycetous yeast genus isolated from plants (Glushakova and Kachalkin 2017b) and soil, with reported *in vitro* antifungal activity against plant-pathogenic *Phytophthora palmivora* (Satianpakiranakorn, Khunnamwong and Limtong 2020)
Basidiomycota	9.35	−5.04 ± 1.02	<0.001	
Entylomatales	5.46	−7.36 ± 2.35	0.002	
Tremellales	4.03	−5.32 ± 1.09	<0.001	
Tremellales	2.70	−3.59 ± 1.34	0.049	
Hypocreales	2.20	−11.87 ± 2.03	<0.001	
Basidiomycota	0.80	−18.02 ± 5.93	0.018	

a Species-level taxa correspond to UNITE species hypotheses (SH).

b Mean normalized count across resistant and susceptible cultivars.

All 19 bacterial OTUs (Table 4) had lower relative abundance in canker-resistant cultivars than in susceptible ones. Of these OTUs, 18 could be assigned to the genus level, including *Sphingomonas* (Sphingomonadaceae, occurring *n* = 2 times), *Massilia* (Oxalobacteriaceae, *n* = 3), *Hymenobacter* (Hymenobacteraceae, *n* = 6), *Frondihabitans* (*n* = 1), *Curtobacterium* (Microbacteriaceae, *n* = 1), *Methylobacterium* (Methylobacteriaceae, *n* = 2), *Pseudomonas* (Pseudomonadaceae, *n* = 2) and *Rathayibacter* (Microbacteriaceae, *n* = 1). The remaining one OTU was assigned to the order Rhizobiales.

Of the 32 fungal taxa with differential abundance (Table 5), eight had higher relative abundance and 23 lower relative abundance in resistant cultivars than in susceptible ones. A heatmap of the average normalized counts of differentially abundant taxa per each scion genotype is shown in Figure S7. Among the eight taxa that showed higher relative abundance in resistant cultivars, four OTUs belonged to Ascomycota, three to Basidiomycota and one was assigned only to the Fungi kingdom. Five OTUs could be identified to the genus rank, including the yeast genera *Rhodotorula* (Sporidiobolaceae, occurring *n* = 1 time) and *Kalmanozyma* (Ustilaginaceae, *n* = 1), as well as the genera of filamentous fungi *Aureobasidium* (Aureobasidiaceae, *n* = 1), *Stemphylium* (Pleosporaceae, *n* = 1) and *Dissoconium* (Dissoconiaceae, *n* = 1). Taxa in the class Dothideomycetes (*n* = 1) and in the order Entylomatales (*n* = 1) were also found.

Among the 23 taxa with lower relative abundance in resistant cultivars, 20 OTUs belonged to the Basidiomycota, one to the Ascomycota and two were assigned only to the Fungi kingdom. Most taxa were found in the class Tremellomycetes (*n* = 16), including the orders Tremellales (*n* = 10) and Filobasidiales (*n* = 5). Within these orders, 13 taxa could be identified at the genus level. The Tremellales included members in the family Bulleribasidiaceae (*Vishniacozyma*, *n* = 2 and *Dioszegia*, *n* = 2), Tremellaceae (*Bulleromyces*, *n* = 1), Phaeotremellaceae (*Gelidatrema*, *n* = 1), Bulleraceae (*Genolevuria*, *n* = 1) and Rhynchogastremataceae (*Papiliotrema*, *n* = 1). The Filobasidiales included members in the genus *Filobasidium* (Filobasidiaceae, *n* = 5). Taxa in the orders Entylomatales (*n* = 1) and Hypocreales (*n* = 1) were also found.

PCA plots and NMDS suggested that the endophyte community structure in ‘Robusta 5’ was highly distinct compared with all other genotypes. Therefore, the relative abundance of bacterial and fungal OTUs was compared between ‘Robusta 5’ and the other scions. Results of DESeq2 differential analysis and the predicted taxonomy for differential OTUs are reported in Table S3 (Supporting Information). Overall, 20 bacterial and 46 fungal OTUs had significantly different relative abundance between ‘Robusta 5’ and the other scion genotypes.

Of the 20 differential bacterial OTUs, one had higher relative abundance and 19 had lower relative abundance in ‘Robusta 5’. The OTU with higher relative abundance was assigned to the genus *Rothia* (Micrococcaceae) but had a very low mean normalized count (0.04). Of the 19 OTUs with lower relative abundance, 16 were also significantly less abundant in resistant genotypes compared with the susceptible (including *Sphingomonas*, *n* = 2; *Curtobacterium*, *n* = 1; *Massilia*, *n* = 1; *Pseudomonas*, *n* = 2; *Hymenobacter*, *n* = 6; *Frondihabitans*, *n* = 1; and *Methylobacterium*, *n* = 2); in addition to these, three more OTUs had lower abundance in ‘Robusta 5’ compared with other genotypes. These could not be assigned at the genus level and had mean normalized count lower than 2.

Of the 46 differential fungal OTUs, 22 had higher relative abundance and 24 had lower relative abundance in ‘Robusta 5’ compared with other genotypes. Of the 22 OTUs with higher relative abundance, 8 were also significantly more abundant in the resistant genotypes compared with the susceptible (including *Rhodotorula*, *n* = 1; *Kalmanozyma*, *n* = 1; *Aureobasidium*,*n* = 1; *Stemphylium*, *n* = 1; and *Dissoconium*, *n* = 1); in addition to these, 14 more OTUs were found with higher abundance in ‘Robusta 5’ compared with all the other genotypes. These included *Alternaria* (*n* = 2), *Sporobolomyces* (*n* = 2), *Periconia* (*n* = 1), *Vishniacozyma* (*n* = 1), *Xanthoria* (*n* = 1), *Preussia* (*n* = 1), *Kondoa* (*n* = 1) and five OTUs that could not be assigned to the genus level (Sporidiobolaceae, *n* = 1; Ascomycota, *n* = 1; Basidiomycota, *n* = 2; Fungi, *n* = 1). Of the 24 OTUs with lower relative abundance, 20 also had lower abundance in the resistant genotypes compared with the susceptible (including Tremellomycetes, *n* = 14; Exobasidiomycetes, *n* = 1; Sordariomycetes, *n* = 1; and four OTUs that could not be assigned at the class level); in addition, four more OTUs had lower relative abundance in ‘Robusta 5’ compared with other genotypes, including *Vishniacozyma* (*n* = 3) and *Genolevuria* (*n* = 1).

## DISCUSSION

In our study, we accounted for large scale spatial effects (i.e. two sites ∼50 km apart), local spatial effects (i.e. within-site blocks), scion genotypes and rootstock genotypes. Both bacterial and fungal communities in apple woody tissues were primarily shaped by the location of the orchard, followed by scion genotypes, agreeing with previous results (Arrigoni *et al*. 2018, 2020; Liu, Ridgway and Jones 2020). Moreover, we found that for both bacteria and fungi a large proportion of the scion effect can be attributed to the differences between canker-resistant and -susceptible cultivars. This upholds our original hypothesis, suggesting that specific components of the overall microbial communities might be associated with, and potentially contribute to, different levels of canker susceptibility. Rootstock effects were very small (bacteria) or negligible (fungi), similar to previous findings (Liu *et al*. 2018).

Location effects are known to shape fungal endophyte community structure in woody and herbaceous plant species on a large scales (i.e. when sampling sites were several km apart), for example in *Theobroma cacao* (Arnold *et al*. 2003), *Cirsium arvense* (Gange *et al*. 2007) and *Impatiens glandulifera* (Currie *et al*. 2020). Location effects on the apple microbiome were also found by Arrigoni *et al*. (2020) and Liu, Ridgway and Jones (2020) in bark and woody stems, respectively. Weather conditions during the week immediately before sampling were comparable across the two sites, with daily mean temperature ranging from 10.3 to 23.1°C in Maidstone and from 10.2 to 23.4°C in Canterbury, and daily mean relative humidity between 61% and 93% in Maidstone and between 53% and 93% in Canterbury. Fungal endophytes appeared to be more affected by the orchard location compared with bacterial endophytes. High-motility airborne inoculum (i.e. fungal conidia and spores) may have contributed to this difference. There was also small-scale within-site effect on bacterial and fungal communities, which may be partially explained by differences in inoculum availability at a very local scale. Ricks and Koide (2019) showed that inoculum dispersal at a local level (i.e. sampling sites were up to 350 m apart) significantly affected the community structure of fungal endophytes associated with the herbaceous plants *Atriplex canescens* and *Bromus tectorum*. In addition, variation in microclimate may also impact the chance of successful establishment at leaf scars. Furthermore, there is small but significant effects of rootstocks in affecting bacterial endophytes in leaf scars. This may suggest that root endophytes may be able to move to above ground organs; hence local variability in bacteria at leaf scar could also partially attributable to local variability in rhizosphere bacteria that could enter into roots.

The amplification of nontarget chloroplast and mitochondrial DNA by the 16S rRNA gene primers utilized in the present study may have reduced the recovery of bacterial sequences (Beckers *et al*. 2016), as indicated by the overall low proportion of bacterial reads after removal of the organelle reads. Additionally, the number of bacterial OTUs found in this study was lower than in other studies. For instance, 513 (Liu *et al*. 2018) and 824 (Arrigoni *et al*. 2018) bacterial OTUs were found in apple stem wood and bark, respectively. However, the accumulation curves showed that sequence depth was satisfactory for all the samples retained in statistical analysis in the present study.

All 18 bacterial genera found with lower relative abundance in resistant scion genotypes are commonly found across a variety of environments, including soil, rhizosphere and plants. All were found in apple, either in stems (Arrigoni *et al*. 2018, 2020; Liu *et al*. 2018), leaves (Yashiro, Spear and McManus 2011), flowers (Shade, McManus and Handelsman 2013) or fruits (Bösch *et al*. 2021). *Pseudomonas* includes both pathogenic (Kennelly *et al*. 2007) and beneficial (Ligon *et al*. 2000; Weller 2007) species. *Sphingomonas* and *Methylobacterium* include species with reported beneficial effects on plants, including plant growth promotion (Dourado *et al*. 2015; Asaf *et al*. 2020) and antagonism against plant pathogens (Wachowska *et al*. 2013; Grossi *et al*. 2020). C*urtobacterium* includes a pathovar pathogenic on herbaceous hosts (Osdaghi *et al*. 2015), as well as species with reported plant growth promoting (Sturz *et al*. 1997) or disease reduction (Lacava *et al*. 2007) activity.

Five of the eight fungal OTUs with higher relative abundance in the canker-resistant cultivars were identified to the genus level. All were previously reported on apple bark (Arrigoni *et al*. 2018) or fruit (Li *et al*. 2012; Weber and Dralle 2013; Bösch *et al*. 2021). The genera *Rhodotorula* and *Kalmanozyma* include species with known biocontrol potential against plant pathogenic fungi. For example, *R. mucilaginosa* had antagonistic activity against the necrotrophic pathogens *Botrytis cinerea* and *Penicillium expansum* on stored apple fruits (Li *et al*. 2011). *K. fusiformata* (syn. *Pseudozyma fusiformata*) was found to produce ustilagic acid, a metabolite with fungicidal activity against a wide range of yeasts and yeasts-like organisms (Golubev, Kulakovskaya and Golubeva 2001). Ustilagic acid also inhibited the growth of the phytopathogenic fungus *Sclerotinia sclerotiorum* (Kulakovskaya *et al*. 2007). *Stemphylium botryosum* and *S. herbarum* cause postharvest rot of apple fruits (Behr 1960; Jijakli and Lepoivre 2004), whereas the causal agent of pear brown spot *S. vesicarium* (Puig *et al*. 2015) was also isolated from blossom-end rot of apple (Weber and Dralle 2013). However, *Stemphylium* sp. was also reported as a hyperparasite on fungi in the family Erysiphales (Sucharzewska *et al*. 2012). Species in the genus *Dissoconium* have been isolated from sooty blotch and flyspeck of apples (Li *et al*. 2012). However, *D. aciculare* also had antagonistic activity against *Erysiphe* spp. causing powdery mildew on different hosts (Crous *et al*. 1999; Kiss 2003).

Interestingly, most fungal taxa with lower relative abundance in the resistant cultivars were Basidiomycetous yeasts in the class Tremellomycetes, orders Tremellales and Filobasidiales. In particular, all endophytes identified to the genus level belonged to these orders. These yeasts have been reported from a wide range of substrates, including forest soil as well as living and decaying plant tissues, and many have been previously reported in apple. Their ecological role is still largely unexplored. However, species in these genera are known, or speculated, to have antifungal activity based on their morphology and physiology (Boekhout *et al*. 2011; Begerow *et al*. 2017). For example, *Bulleromyces albus* (anamorph: *Bullera alba*) had antifungal activity against different species of Ascomycota and Basidiomycota (Golubev *et al*. 1997), and haustoria were observed in *Dioszegia* spp., suggesting a mycoparasitic lifestyle (Connell *et al*. 2010). Antagonistic activity has been reported for *P. laurentii* against *Phytophthora palmivora* (Satianpakiranakorn, Khunnamwong and Limtong 2020), whereas *V. victoriae* reduced postharvest decay of pears caused by *B. cinerea* and *P. expansum* (Lutz *et al*. 2020).

Fungal endophytes with higher relative abundance in resistant genotypes and reported antifungal activity, either by mycoparasitism or production of toxic metabolites, might directly antagonize *N. ditissima*, alone or in synergy with each other or other microbial species. Although some of these are known as plant pathogens, ecological functions are considered context-dependent (Newton *et al*. 2010; Busby, Ridout and Newcombe 2016). The same microorganism might behave as an aggressive pathogen or a beneficial mutualist, or remain neutral, based on factors determined by the host (i.e. the genotype and the development stage), the abiotic environment or the biotic environment (i.e. presence of other pathogens and plant-associated microorganisms). Liu, Ridgway and Jones (2020) isolated 19 bacterial and 17 fungal endophytes from young stems and leaves of different apple cultivars, all showing antagonistic activity against *N. ditissima*. Some of these endophytes correspond to species (*Phlyctema vagabunda*, syn. *Neofabraea alba*), or genera (*Diaporthe*) including species with pathogenic lifestyle on apple. The same may apply to the fungal endophytes found with higher relative abundance in resistant apple genotypes in the present study. On the other hand, fungal and bacterial genera with lower relative abundance in resistant genotypes in this study might represent pathogen facilitators for *N. ditissima*. In particular, the high prevalence of tremellomycetous yeasts in this group suggests that they may compete with fungal antagonists of the pathogen. Competition between Tremellomycetes and a biocontrol agent of a fungal pathogen has been proposed by Vujanovic (2021). Following treatment of wheat (*Triticum turgidum*) plants with *Sphaerodes mycoparasitica*, a biotrophic mycoparasite of *Fusarium graminearum* (Fusarium head blight, FHB), lower incidence and severity of the disease, as well as lower relative abundance of *Vishniacozyma* spp. in kernels, compared with the control, were recorded. Conversely, when plants were treated with a different plant growth promoting fungus (*Penicillium* sp.), incidence and severity of FHB remained high and higher relative amount of *Vishniacozyma* spp. in kernels was observed. Interestingly, Griffiths *et al*. (2019) reported that *Genolevuria* sp. was significantly more abundant in leaves of *Fraxinus excelsior* (European ash) infected with *Hymenoscyphus fraxineus* (ash dieback), compared with healthy leaves. Together with these findings, the present results suggest that tremellomycetous yeasts might play an important role as disease modifiers in different plant pathosystems.

To gain further insight into the ecological functions of endophytes in the *N. ditissima* pathosystem, experiments could be designed to study endophyte population dynamics following or not inoculation with the pathogen, in different apple genotypes. In fact, the presence of the pathogen may cause shifts in endophyte composition and relative abundance, which could especially affect potential pathogen antagonists or facilitators. These shifts may result from the direct interaction between the pathogen and the endophytes. However, they could also be mediated by the host genotype, as a component of the response to *N. ditissima* infection, and therefore differ across canker-resistant and -susceptible cultivars. To understand how endophytes could be harnessed to control *N. ditissima*, specific endophytes could be isolated using selective media and growth conditions and used in manipulation studies. Interactions between endophytes, host and pathogen can be greatly influenced by biotic and abiotic factors, including for example the order of arrival of different inocula on the plant (Adame-Álvarez, Mendiola-Soto and Heil 2014) or the host genotype (Gange *et al*. 2007). Therefore, manipulation experiments may achieve more consistent results by employing microbial consortia instead of individual microorganisms, and by studying their effect *in planta* rather than *in vitro*. The relative abundance of endophytes across the apple genotypes in this present work could help select specific endophytes and inform their respective doses in complex inocula.

Among the different genotypes, ‘Robusta 5’ hosted the most distinctive fungal and bacterial endophyte community, and clustered separately from all other genotypes in the PCA and the NMDS plots. This genotype, *Malus* × *robusta*, represents a distinct species from the other genotypes used in the study, which were all *M*. × *domestica* accessions. Thus, it is possible that genotypic differences between ‘Robusta 5’ and all other scion cultivars used in this study contributed to the marked differences in endophyte community structure. Forsline *et al*. (2003) described ‘Robusta 5’ as a hybrid between *M. baccata* and *M. prunifolia*, and much plant material of this cultivar in Europe has been imported from Northern China (Gomez-Cortecero *et al*. 2016). ‘Robusta 5’ was found highly resistant to *N. ditissima* (Gomez-Cortecero *et al*. 2016), and a quantitative trait locus controlling the resistance trait has been recently identified (Bus *et al*. 2019). In the present study, bacterial genera with lower relative abundance in ‘Robusta 5’ also had lower abundance in the resistant cultivar group, except for *Rathayibacter*. Conversely, *Rothia* had higher relative abundance in ‘Robusta 5’ but not in the resistant cultivar group. Endophytic species were reported in this genus (Borah and Thakur 2020). Fungal OTUs with higher relative abundance in ‘Robusta 5’ compared with other cultivars included all OTUs with higher abundance in the resistant cultivar group, plus several additional OTUs assigned to genera including species with antifungal activity, namely *Alternaria*, *Sporobolomyces*, *Vishniacozyma*, *Periconia*, *Preussia* and *Xanthoria*. Endophytic *Alternaria* spp. from coniferous trees (Cupressaceae) inhibited *in vitro* growth of the plant pathogenic fungi *Diplodia seriata*, *Phaebotryon cupressi* and *Spencermartinsia viticola* (Soltani and Hosseyni Moghaddam 2014), whereas *Sporobolomyces roseus* efficiently antagonized *B. cinerea* and *P. expansum* on stored apple fruits (Janisiewicz, Peterson and Bors 1994). Activity against plant pathogenic fungi is also documented *in vitro* for *Periconia* sp. (Luo *et al*. 2015) and for extracts of *Preussia* sp. (Gherbawy and Elhariry 2016) and the lichen *Xanthoria parietina* (Basile *et al*. 2015). Finally, fungal OTUs with lower relative abundance in ‘Robusta 5’ compared with the other cultivars were assigned to the same genera in the class Tremellomycetes as those found with lower relative abundance in the resistant cultivar group, except for *Papiliotrema*. The present findings suggest that this genotype may harbour a higher number of potential *N. ditissima* antagonists, compared with the other cultivars used in this study, thus representing a promising source of active isolates. In addition, the present results may lend further support to the hypothesis that Tremellomycetes hold important disease modification functions in the apple canker pathosystem.

In conclusion, we found that a significant proportion of the diversity of apple endophytes at leaf scars was associated with the cultivar susceptibility to *N. ditissima*. Moreover, we identified specific bacterial and fungal genera with differential relative abundance in canker-resistant genotypes of apple compared with susceptible ones. Most fungal genera with differential abundance included members with known—or predicted—antifungal activity. The present results may be used to inform targeted approaches to further the research in *N. ditissima* biological control.

## ACKNOWLEDGEMENTS

We are grateful to the Agriculture and Horticulture Development Board (AHDB) for funding this work within the project CP161: Understanding Endophytes to Improve Tree Health. We thank Nick Dunn at Frank P Matthews, Tenbury Wells, Worcestershire, UK for carrying out grafting and storing the trees until planting. We also thank Jack Skinner at William Skinner & Son, Maidstone, UK and Peter Chandler at Chandler & Dunn, Canterbury, UK for providing the planting sites and carrying out orchard management operations throughout the experiment. We are also grateful to Nigel Jenner at Avalon Produce Ltd and Tony Harding at Worldwide Fruit Ltd for partnering the project and coordinating grower sites. We are grateful to Dr Tom Passey for his help in planting trees, arranging orchard inspections, collecting plant material, preparing the sequencing library and running the sequencing with Illumina MiSeq. We thank Dr Greg Deakin for his help with the bioinformatics and statistical analysis. We also thank Dr Matevs Papp-Rupar and Dr Lucas Shuttleworth for their help in collecting plant material and preparing samples for DNA extraction.

## Supplementary Material

fiab131_Supplemental_Files
