# Supplementary material for: Apple endophyte community in relation to location, scion and rootstock genotypes and susceptibility to European canker
Source: FEMS Microbiol Ecol. 2021 Oct 2;97(10):fiab131. doi: 10.1093/femsec/fiab131 (PMC8497447; doi:10.1093/femsec/fiab131)
Supplement: fiab131_Supplemental_Files [file fiab131_supplemental_files.zip › Supplementary_Data_descriptions_reviewed.docx]

**Table S1** – Alpha (α) diversity indices for bacteria and fungi in resistant and susceptible cultivars.

**Table S2** – Best prediction of taxonomy, using UTAX and BLASTn, for fungal and bacterial OTUs with differential abundance between resistant and susceptible cultivar groups.

**Table S3** – Best prediction of taxonomy, using UTAX and BLASTn, for fungal and bacterial OTUs with differential abundance between ‘Robusta 5’ and all other scion cultivars.

**Figure S1** – Sample accumulation curves for 16S reads.

**Figure S2** – Overall distribution read counts for bacterial OTUs.

**Figure S3** – Sample accumulation curves for ITS reads.

**Figure S4** – Overall distribution read counts for fungal OTUs

**Figure S5** – Alpha (α) diversity indices for bacteria and fungi in resistant and susceptible cultivars at the two sites of planting.

**Figure S6** – Plot showing Log_2_ of DESeq2 normalised count vs Log_2_ fold change of bacterial and fungal OTU counts.

**Figure S7** - Heatmap showing the average normalised counts of differentially abundant taxa across all scion genotypes.
