# Supplementary material for: Apple endophyte community in relation to location, scion and rootstock genotypes and susceptibility to European canker
Source: FEMS Microbiol Ecol. 2021 Oct 2;97(10):fiab131. doi: 10.1093/femsec/fiab131 (PMC8497447; doi:10.1093/femsec/fiab131)
Supplement: fiab131_Supplemental_Files [file fiab131_supplemental_files.zip › Supplementary_Data_Table_S1.docx]

| **Supplementary Table 1** - Alpha (α) diversity indices (observed species, Chao1, Simpson and Shannon) and respective standard error (SE) for bacterial and fungal normalised OTU counts from resistant and susceptible cultivars. Shannon index was calculated using the natural logarithm. | | | | |
| --- | --- | --- | --- | --- |
| **Group** | **Index** | | | |
|  | **Observed ±SE** | **Chao1 ±SE** | **Shannon ±SE** | **Simpson ±SE** |
| *Bacteria* |  |  |  |  |
| Resistant | 21.63 ±2.29 | 33.06 ±4.01 | 1.97 ±0.11 | 0.74 ±0.03 |
| Susceptible | 24.77 ±1.40 | 35.56 ±2.30 | 2.29 ±0.04 | 0.83 ±0.01 |
| *Fungi* |  |  |  |  |
| Resistant | 97.63 ±6.11 | 142.10 ±15.65 | 2.35 ±0.04 | 0.83 ±0.01 |
| Susceptible | 128.65 ±3.73 | 248.15 ±23.06 | 2.44 ±0.03 | 0.84 ±0.00 |
