# Supplementary figures and images for: Apple endophyte community in relation to location, scion and rootstock genotypes and susceptibility to European canker
Source: FEMS Microbiol Ecol. 2021 Oct 2;97(10):fiab131. doi: 10.1093/femsec/fiab131 (PMC8497447; doi:10.1093/femsec/fiab131)

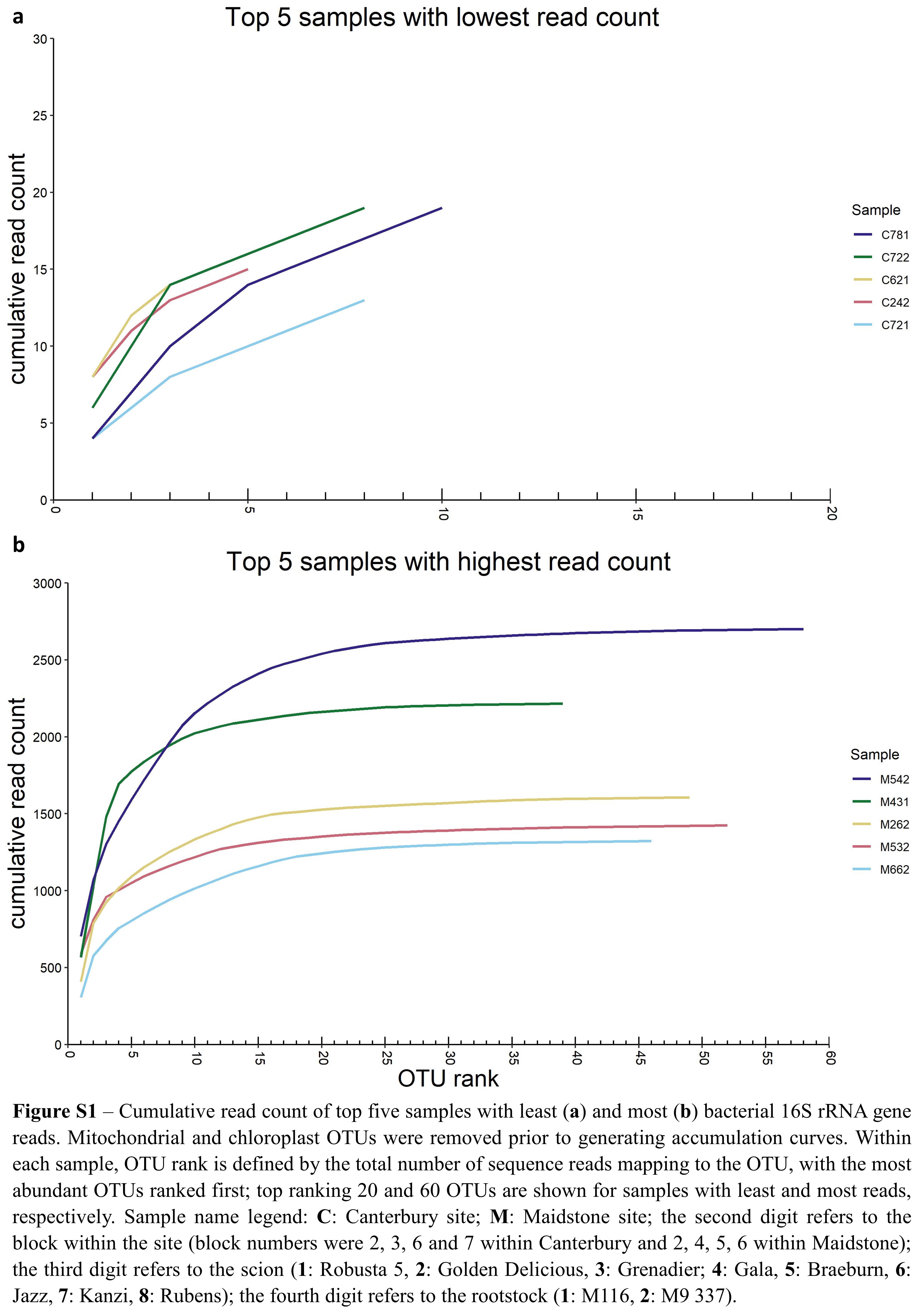

Supplement: fiab131_Supplemental_Files [file fiab131_supplemental_files.zip › Supplementary_Data_Figure_S1_reviewed_v2.jpg]

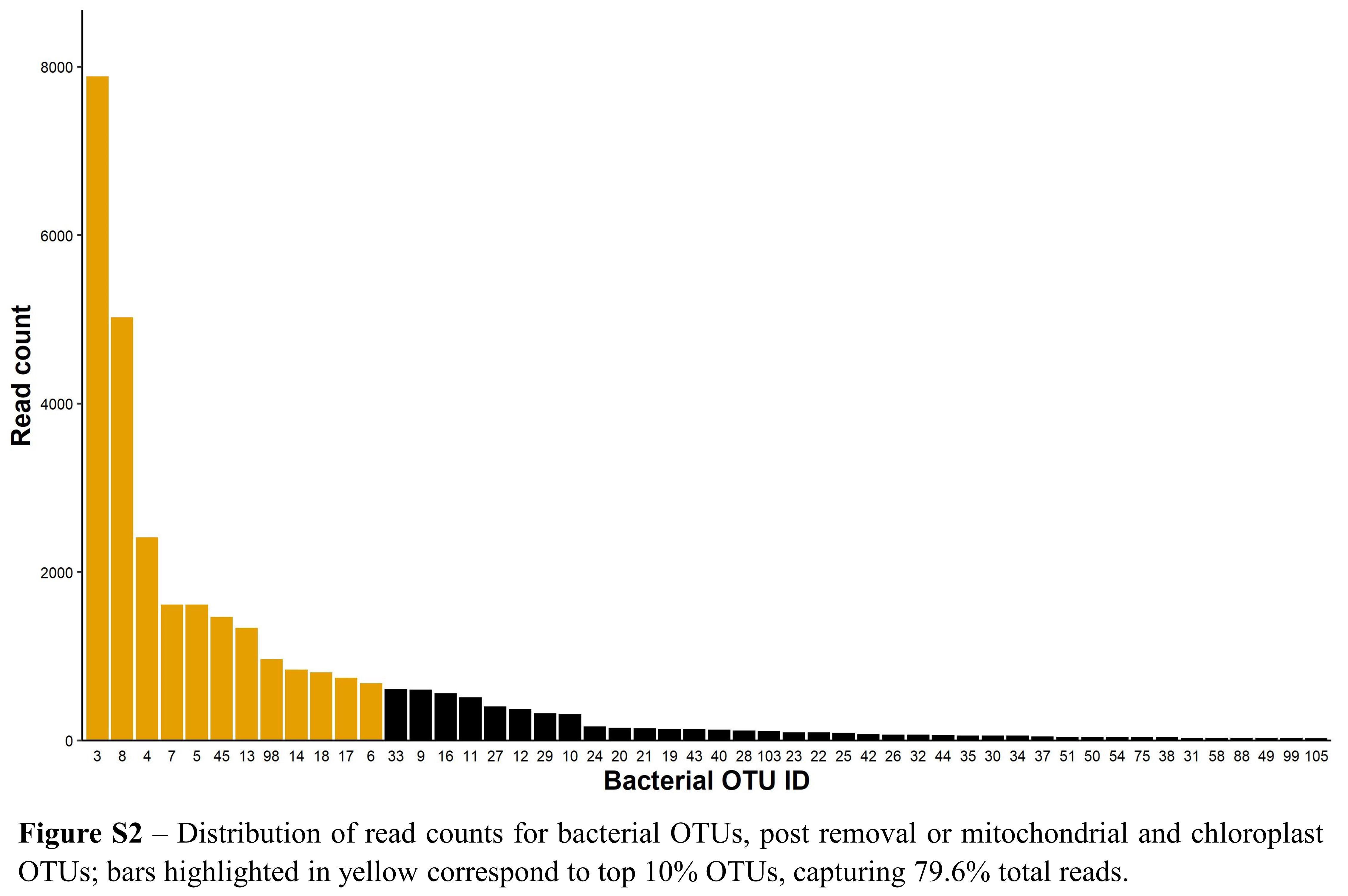

Supplement: fiab131_Supplemental_Files [file fiab131_supplemental_files.zip › Supplementary_Data_Figure_S2_600dpi.jpg]

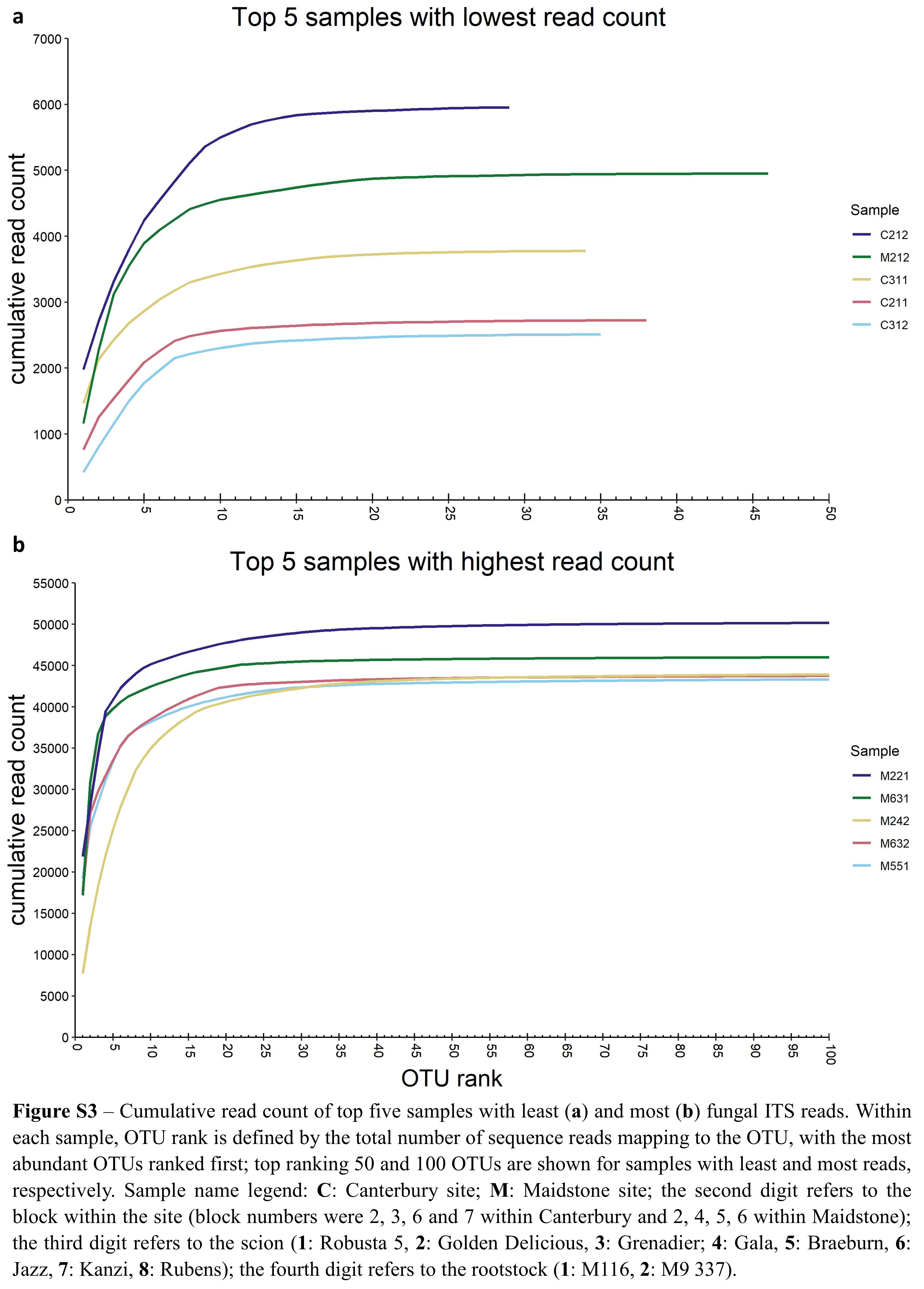

Supplement: fiab131_Supplemental_Files [file fiab131_supplemental_files.zip › Supplementary_Data_Figure_S3_reviewed.jpg]

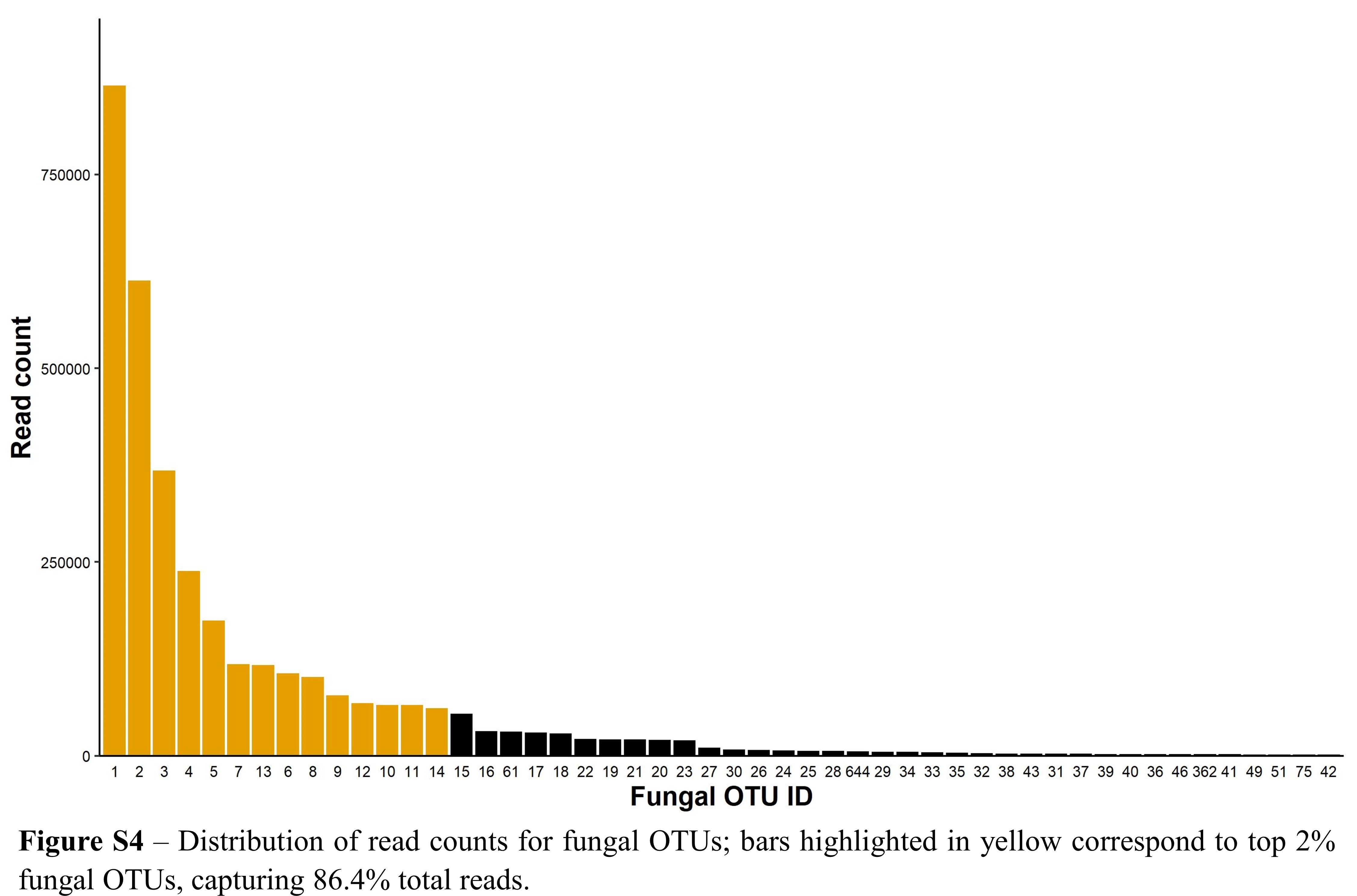

Supplement: fiab131_Supplemental_Files [file fiab131_supplemental_files.zip › Supplementary_Data_Figure_S4_600dpi.jpg]

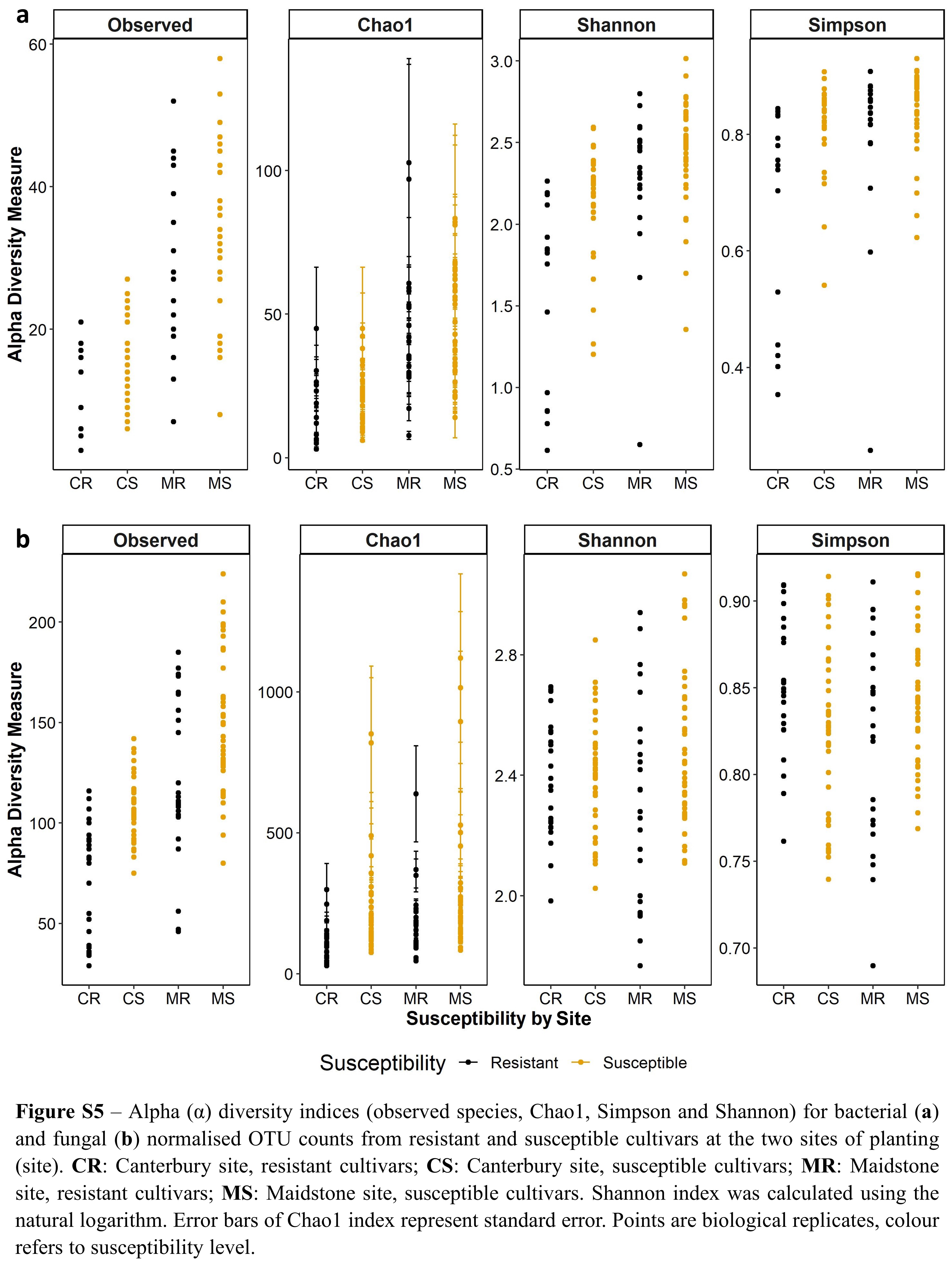

Supplement: fiab131_Supplemental_Files [file fiab131_supplemental_files.zip › Supplementary_Data_Figure_S5_reviewed.jpg]

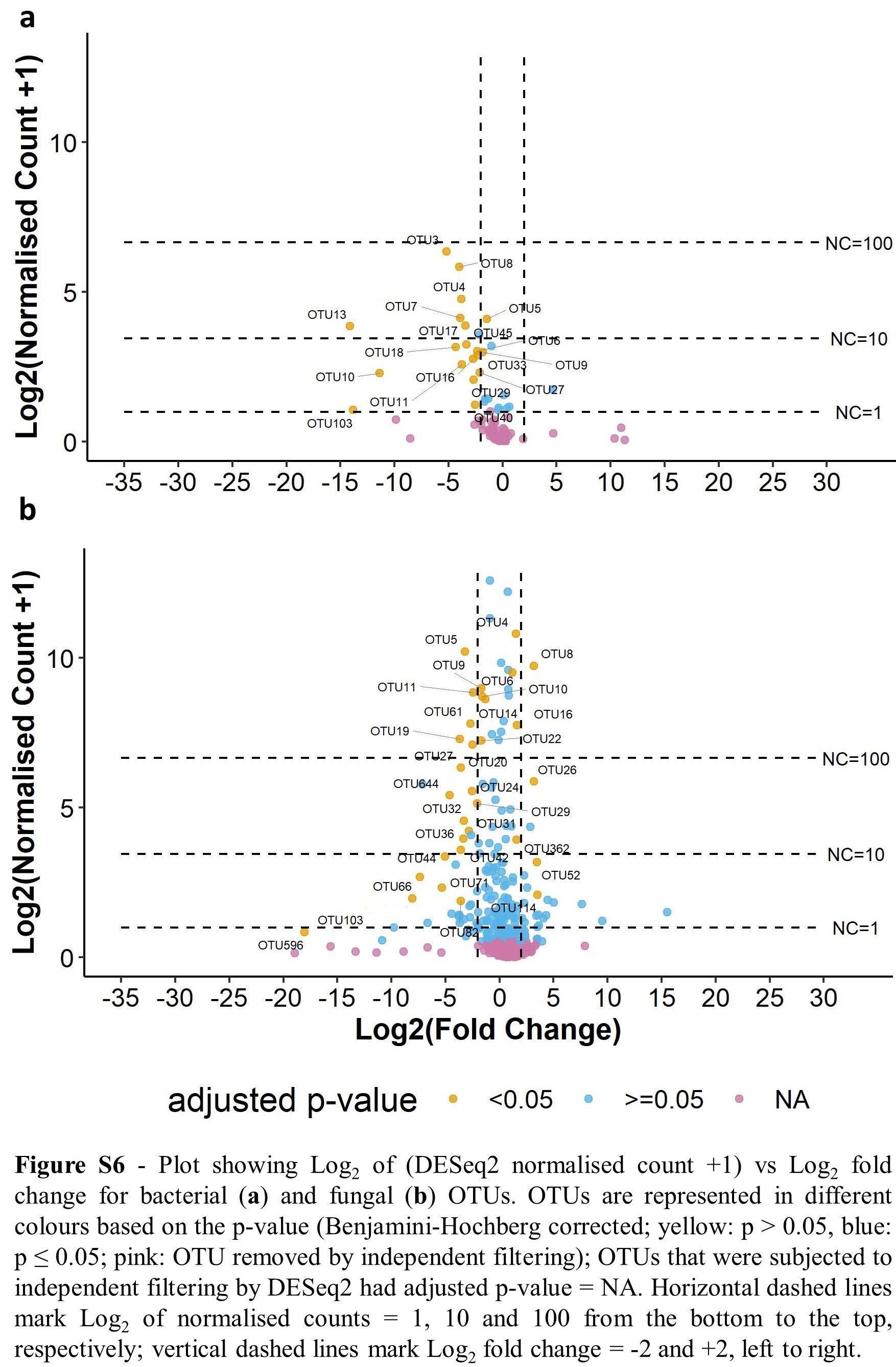

Supplement: fiab131_Supplemental_Files [file fiab131_supplemental_files.zip › Supplementary_Data_Figure_S6_reviewed.jpg]

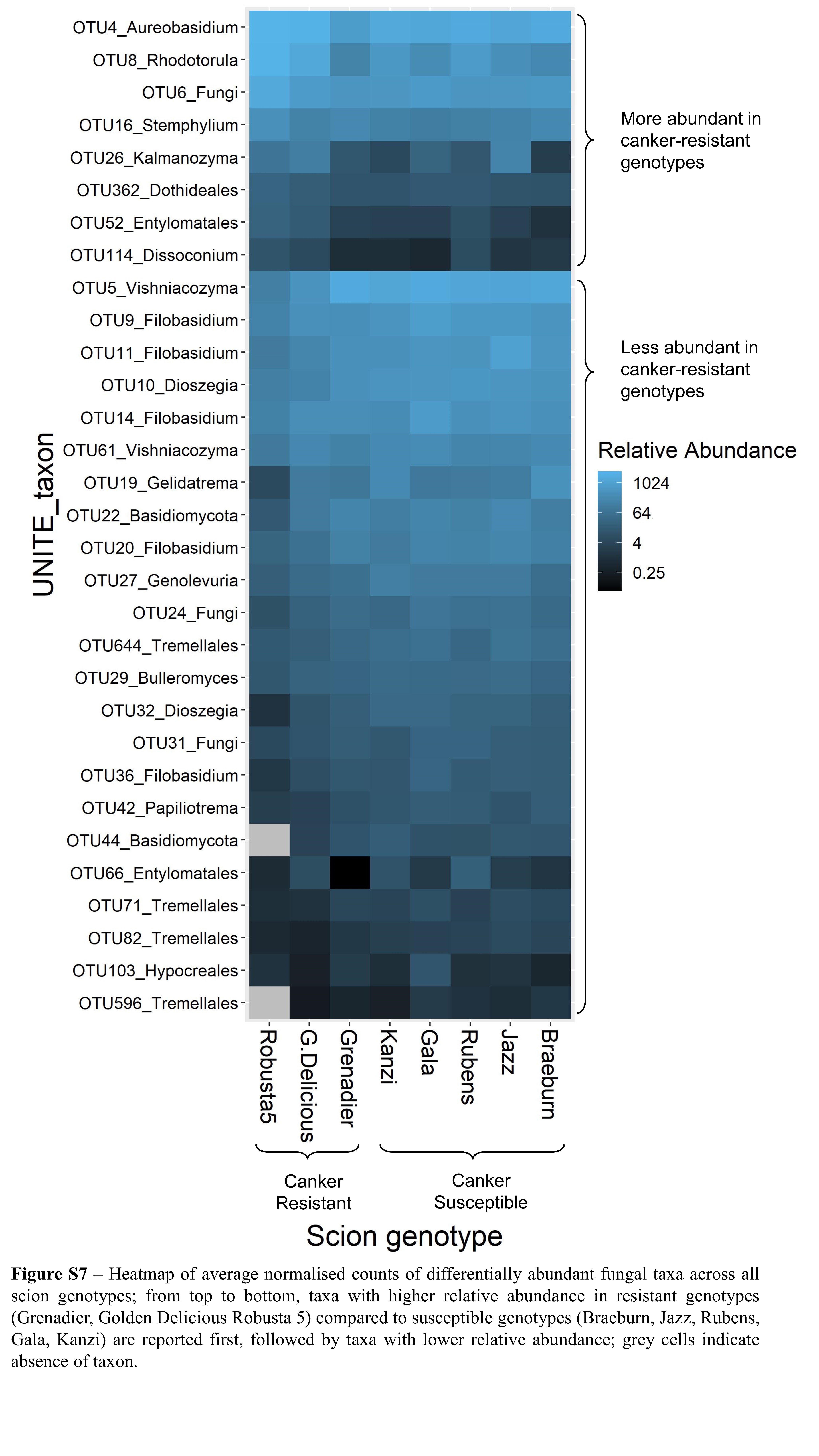

Supplement: fiab131_Supplemental_Files [file fiab131_supplemental_files.zip › Supplementary_Data_Figure_S7_reviewed.jpg]
